# Special Issue “Post-COVID-19 Symptoms in Long-Haulers: Definition, Identification, Mechanisms, and Management”

**DOI:** 10.3390/jcm12206458

**Published:** 2023-10-11

**Authors:** César Fernández-de-las-Peñas, Domingo Palacios-Ceña

**Affiliations:** Department of Physical Therapy, Occupational Therapy, Physical Medicine and Rehabilitation, Universidad Rey Juan Carlos (URJC), 28922 Alcorcón, Spain; domingo.palacios@urjc.es

The worldwide spread of coronavirus disease 2019 (COVID-19), a condition caused by the severe acute respiratory syndrome coronavirus 2 (SARS-CoV-2) pathogen, led to the most unprecedented disease outbreak of this century, provoking around 770 million confirmed cases and nearly 7 million deaths globally [1]. The extensive literature about COVID-19 has concentrated on the disease and the management of acute cases [2]. With the development of COVID-19 vaccines, hundred of studies investigating their effects have been published [3].

Despite incommensurable efforts being exerted in fighting against SARS-CoV-2, the world is now faced with a post-COVID-19 pandemic, the “long-haulers”, individuals who recovered from COVID-19 but developed long-lasting symptomatologies or post-COVID-19 conditions [4]. Evidence describes the presence of up to 100 post-COVID-19 symptoms [5]. The Global Burden of Disease Long COVID study which included 1.2 million of subjects who had experienced an acute symptomatic SARS-CoV-2 infection, reported that up to 15.1% of COVID-19 survivors experienced long-lasting symptoms one year after infection [6]. Rahmati et al., reported that 41.7% of COVID-19 survivors still experienced post-COVID-19 symptoms two years after infection [7]. However, gaps exist in definitions, timeframe, identification, mechanisms, and treatment strategies for the management of these post-COVID-19 symptoms [8]. The aim of this Special Issue entitled “Post-COVID-19 Symptoms in Long-Haulers: Definition, Identification, Mechanisms, and Management” of the *Journal of Clinical Medicine* focuses on different aspects of post-COVID-19 symptoms, a topic of emerging relevance due to the expected presence of millions of “long-haulers”. Herein, an overview of all the papers included in this Special Issue is provided.

One study investigated sex differences in the development of COVID-19-associated onset symptoms and post-COVID-19 symptoms [9]. The authors found similar onset symptoms but a higher number of post-COVID-19 symptoms in females than in males [9]. Thus, female sex was associated with a higher presence of post-COVID-19 fatigue, dyspnea, pain, hair loss, and ocular problems, as well as higher depressive levels and worse sleep quality [9]. The fact that female sex is a risk factor associated with post-COVID-19 symptoms has been confirmed in two posterior meta-analyses [10,11]. Biological and psychological sex differences could explain the higher risk for females [12,13,14], which should be considered in future studies where post-COVID-19 treatment would be conducted from a sex perspective.

Fatigue, brain fog and pain are among the most prevalent post-COVID-19 symptoms [6,7]. Post-COVID-19 pain is experienced by 15–20% of subjects after an acute SARS-CoV-2 infection [15,16]. Two papers published in this Special Issue investigated different aspects of pain symptomatology, since the identification of potential risk factors associated with specific post-COVID-19 symptoms can help clinicians to better understand this condition. Both papers focused on the presence of sensitization-associated symptoms in people with post-COVID-19 pain. Several musculoskeletal chronic pain conditions are associated with sensitization [17]. In fact, the evidence supports the presence of sensitization symptoms in individuals with post-COVID-19 condition to a similar extent to in people with fibromyalgia syndrome [18]. Since one clinical manifestation of pain sensitization is the presence of larger areas of pain [19], its association with other measures of central sensitization could reveal potential mechanisms of post-COVID-19 pain. In individuals with post-COVID-19 pain, the pain extent was not associated either with psychophysical (e.g., pressure pain thresholds) or with psychological (anxiety, depression, or sleep quality) outcomes, suggesting that pain extent represents a different aspect of the pain spectrum [20]. Similarly, the presence of sensitization-associated symptoms was not associated with serological biomarkers at hospital admission in a cohort of previously hospitalized COVID-19 survivors [21]. These results suggest that sensitization-associated symptoms and their relevance in patients with post-COVID-19 pain need to be further investigated, since it would be possible that a group of individuals with post-COVID-19 pain exhibit a nociplastic phenotype [22] and, hence, require particular attention. In fact, current hypotheses suggest that post-COVID-19 conditions and fibromyalgia syndrome share common mechanisms [23].

The study by Matias-Guiu et al., tried to identify cognitive predictors of the most prevalent post-COVID-19 symptom, fatigue [24]. This study was not able to identify any neuropsychological predictor explaining the presence of post-COVID-19 fatigue, suggesting that these symptoms, i.e., fatigue and cognitive impairments [24]. This study suggests that physical and cognitive post-COVID-19 symptoms exhibit different underlying pathophysiological mechanisms [24].

The remaining papers included specific populations. For instance, Yamamoto et al., identified the presence of late-onset hypogonadism in almost 50% of individuals who had survived COVID-19 [25]. This study found that the association between the serum level of free testosterone and levels of blood hemoglobin and serum total protein and albumin observed in COVID-19 survivors was not identified in individuals developing late-onset hypogonadism [26]. Another study including kidney transplant recipients, a vulnerable population, identified that 70% developed post-COVID-19 symptoms, with fatigue, hair loss, memory impairment and pain being the most prevalent symptoms [26]. The development of post-COVID-19 symptoms in this population is highly relevant, since kidney transplant recipients show a deficient antibody immune response after the administration of an mRNA SARS-CoV-2 vaccine [27].

The last paper published aimed to identify the role of endothelial dysfunction and the reduced cardiopulmonary exercise performance exhibited by COVID-19 survivors [28]. This study found that alteration of the endothelial barrier properties in systemic and pulmonary circulation may represent a key mechanism of the reduced cardiopulmonary exercise performance identified in this population [28]. The identification of an alteration of endothelial barrier properties could lead to better rehabilitation strategies targeting this mechanism and could explain why some patients with a post-COVID-19 condition develop post-exertional malaise. Again, this endothelial dysfunction is also observed in individuals with myalgic encephalomyelitis/chronic fatigue syndrome [29,30], another entity showing common mechanisms with post-COVID-19 conditions [31].

As Guest Editors of this Special Issue, we would like to thank the reviewers for their insightful comments, all authors for their valuable contributions, and the *JCM* staff for their collective support and assistance during this process.
